# Structural basis of ELKS/Rab6B interaction and its role in vesicle capturing enhanced by liquid-liquid phase separation

**DOI:** 10.1016/j.jbc.2023.104808

**Published:** 2023-05-11

**Authors:** Gaowei Jin, Leishu Lin, Kaiyue Li, Jiashan Li, Cong Yu, Zhiyi Wei

**Affiliations:** 1 Brain Research Center, Southern University of Science and Technology, Shenzhen, Guangdong, China; 2 School of Life Sciences, Southern University of Science and Technology, Shenzhen, Guangdong, China; 3 Guangdong Provincial Key Laboratory of Cell Microenvironment and Disease Research, Shenzhen Key Laboratory of Cell Microenvironment, Shenzhen, Guangdong, China

**Keywords:** intracellular transport, active zone, CAST, Rab6A, BICD, protein-protein interaction, Rab-binding domain, biological condensate, cargo unloading, neuropeptide secretion

## Abstract

ELKS proteins play a key role in organizing intracellular vesicle trafficking and targeting in both neurons and non-neuronal cells. While it is known that ELKS interacts with the vesicular traffic regulator, the Rab6 GTPase, the molecular basis governing ELKS-mediated trafficking of Rab6-coated vesicles, has remained unclear. In this study, we solved the Rab6B structure in complex with the Rab6-binding domain of ELKS1, revealing that a C-terminal segment of ELKS1 forms a helical hairpin to recognize Rab6B through a unique binding mode. We further showed that liquid-liquid phase separation (LLPS) of ELKS1 allows it to compete with other Rab6 effectors for binding to Rab6B and accumulate Rab6B-coated liposomes to the protein condensate formed by ELKS1. We also found that the ELKS1 condensate recruits Rab6B-coated vesicles to vesicle-releasing sites and promotes vesicle exocytosis. Together, our structural, biochemical, and cellular analyses suggest that ELKS1, *via* the LLPS-enhanced interaction with Rab6, captures Rab6-coated vesicles from the cargo transport machine for efficient vesicle release at exocytotic sites. These findings shed new light on the understanding of spatiotemporal regulation of vesicle trafficking through the interplay between membranous structures and membraneless condensates.

Intracellular vesicle trafficking is a complex and tightly controlled process that involves the transport of membrane-bound vesicles between different cellular compartments (1, 2, 3). The proper targeting of these vesicles to their destinations is essential for many cellular activities, including neurotransmitter release, hormone secretion, and intracellular signaling (1, 4). In neurons, for example, synaptic vesicles must be unloaded at the presynaptic terminal in a highly regulated manner to ensure proper synaptic transmission (5, 6), which requires communication between vesicle-associated proteins and proteins functioning at the vesicle-releasing site.

Rab6 is an evolutionarily conserved Rab-GTPase that associates with vesicles and regulates vesicle trafficking to and from the Golgi apparatus (7, 8, 9, 10). Among mammalian Rab6 proteins, Rab6A and Rab6B which share a high sequence similarity (>90% sequence identity) have been extensively studied (11). Rab6A is ubiquitously expressed while Rab6B is predominantly expressed in the brain tissue (12, 13). Recent research showed that Rab6B depletion leads to defects of axonal cargo delivery and aberrates the synaptic vesicle deposition in the presynapse (14). Several Rab6 effectors, like Rab6IP1, GCC185, and KIF20A, have also been reported to regulate Rab6-mediated vesicle trafficking at the Golgi. Considering that Rab6-positive vesicles are transported together with motor proteins and effectors (15, 16), it is still puzzling how these vesicles are unloaded from the transport machine and retained at the secretion site.

As Rab6 effectors, the scaffold proteins ELKS (also known as Rab6IP2, CAST, or ERC) in vertebrates are encoded by two genes, *Erc1* and *Erc2*, expressing ELKS1 and ELKS2, respectively (17, 18, 19, 20). Unlike other Rab6 effectors that function predominantly in Golgi-related processes, ELKS proteins play a crucial role in organizing the deposition and secretion of Rab6-positive vesicles in both neuronal and nonneuronal cells (21). In neurons, ELKS proteins are localized to the presynaptic active zone, where they provide molecular scaffolding by interacting with other active zone proteins (*e.g.*, liprin-α and RIM) for regulating synaptic vesicle unloading and neurotransmitter release (22, 23). In HeLa cells, ELKS1 enriches the cortical sites with membrane-anchored protein LL5β and forms a platform for constitutive vesicle secretion (24). In pancreatic β-cells, ELKS is important for efficient insulin exocytosis by defining the vesicle fusion site of insulin granules (25, 26). The ELKS proteins consist of an N-terminal intrinsic disorder region (IDR), a middle long coiled-coil region and a C-terminal Postsynaptic density-95/Discs large/Zona occludens-1 (PDZ)-binding motif (PBM) (Fig. 1*A*). The Rab6-binding site has been mapped to a C-terminal part of the coiled-coil region in ELKS (14, 18). However, the molecular mechanism underlying the ELKS-mediated Rab6-coated vesicle trafficking remains elusive.

**Figure 1:**
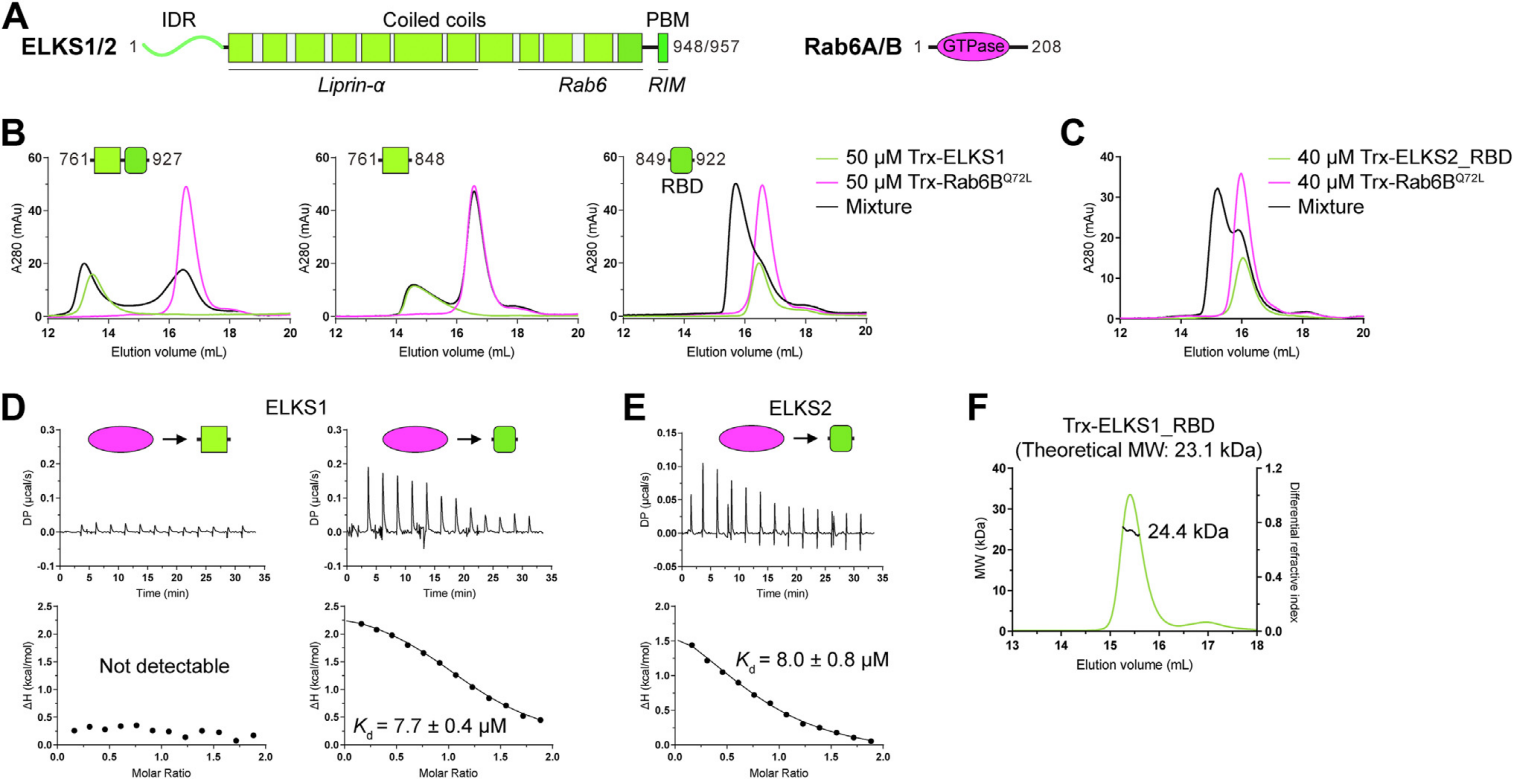
**Biochemical characterization of the interaction between ELKS and Rab6B.***A*, schematic diagrams showing the domain organizations of ELKS and Rab6. The protein interacting regions in ELKS were indicated. The color coding for proteins was used throughout the entire manuscript except as otherwise indicated. *B*, analytical gel filtration chromatography shows that ELKS1_RBD contains the necessary region for binding to Rab6B. *C*, analytical gel filtration chromatography showing the interaction between ELKS2 and the active form of Rab6B. *D*, ITC-based analyses confirming that Rab6B^Q72L^ binds to ELKS1_RBD but not the coiled-coil region at the N-terminal to the RBD. *E*, ITC-based analysis confirming the ELKS2_RBD/Rab6B^Q72L^ interaction. *F*, multiangle light static scattering analysis indicating that the ELKS1_RBD forms a monomer in solution. ITC, Isothermal titration calorimetry.

To better understand the structural basis of the ELKS/Rab6 interaction and its role in vesicle trafficking and targeting, we biochemically characterized the interaction between ELKS1 and Rab6B and determined the atomic structure of the Rab6-binding domain of ELKS1 in complex with the constitutively active Rab6B mutant, Rab6B^Q72L^. Our structural analyses revealed a unique Rab6-binding mode for ELKS by comparison to other Rab/effector interactions. In addition, we found that Rab6-coated vesicles can be recruited to the ELKS1 condensates formed *via* LLPS. Disrupting the ELKS1/Rab6 interaction or the condensate formation of ELKS1 led to the defective secretion of neuropeptide Y carried by Rab6-positive vesicles. These results suggest that by capturing Rab6-coated vesicles, ELKS facilitates the unloading of cargo from the transport machine and promotes their secretion.

## Results

### Mapping the minimal Rab6-binding domain in ELKS1 and ELKS2

To gain insights into the role of ELKSs in the trafficking of Rab6-coated vesicles, we set out to characterize the binding mode between ELKSs and Rab6. We purified the C-terminal coiled-coil region (residues 761–927) of ELKS1 and the GTPase domain of Rab6B (Fig. 1*A*). In agreement with the previous GST-pull down assay (14), our analytical gel filtration analysis showed that the C-terminal fragments of ELKS1 and ELKS2 bind to the continuously active mutant Rab6B^Q72L^ but not the inactive mutant Rab6B^T27N^ (Figs. 1*B* and S1). The Rab6-binding site in ELKS1 was further narrowed down to the C-terminal region containing residues 849 to 922 (Fig. 1*B*), named the Rab-binding domain or RBD hereafter. Similarly, the corresponding region in ELKS2 (ELKS2_RBD) also formed the complex with Rab6B^Q72L^ in the analytical gel filtration (Fig. 1*C*).

To measure the binding affinity between ELKS_RBDs and Rab6B, we performed isothermal titration calorimetry (ITC)-based experiments. As indicated in Figure 1, *D* and *E*, ELKS1_RBD and ELKS2_RBD bind to Rab6B^Q72L^ with a similar affinity of ∼8 μM, whereas the coiled-coil at the N-terminal to ELKS1_RBD showed no detectable binding to Rab6B^Q72L^, consistent with the analytical gel filtration analysis. Surprisingly, despite the location of the RBD in the predicted coiled-coil region, the molecular weight (MW) of the Trx-tagged ELKS1_RBD protein was measured to be ∼24 kDa using analytical gel filtration chromatography coupled with multi-angle static light scattering (Fig. 1*F*). This measured MW is close to the theoretical MW of the monomeric form, indicating that ELKS1_RBD may not form a dimeric coiled-coil and instead exists as a monomer in solution.

### Structural analyses of the ELKS1_RBD/Rab6B^Q72L^ complex

**Figure 2:**
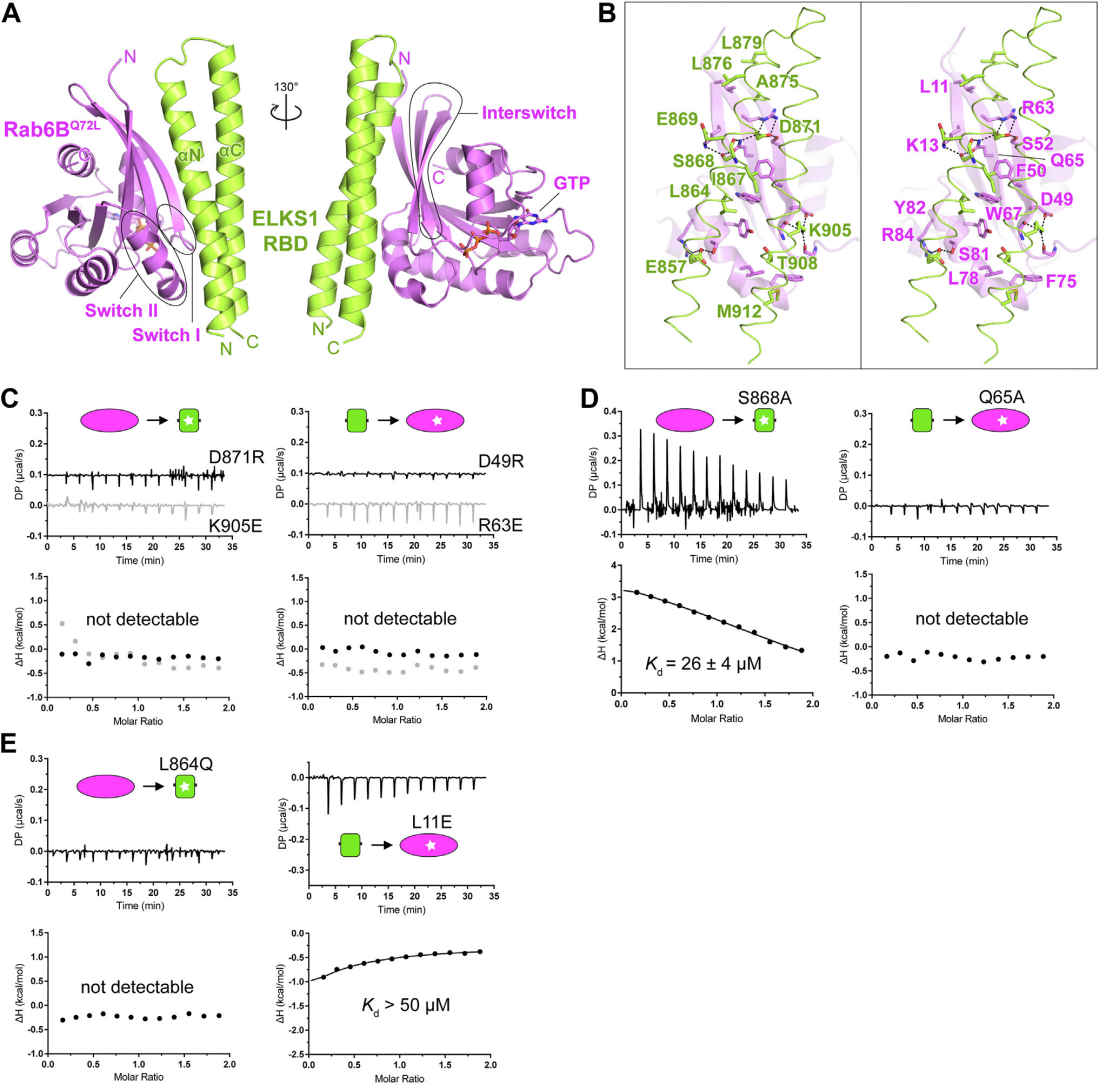
**Structural analysis of the interaction between ELKS1 and Rab6B**^**Q72L**^**.***A*, the overall structure of the ELKS1_RBD/Rab6B^Q72L^ complex. The switch I/II and interswitch regions of Rab6B were indicated. *B*, molecular details of the ELKS1_RBD/Rab6B^Q72L^ interaction with interface residues highlighted in *sticks*. Hydrogen bonds and salt bridges were indicated by *dashed lines*. *C–E*, ITC-based analyses of the disruptive interactions between ELKS1_RBD and Rab6B^Q72L^ mutants, including the mutations of interface residues that interfere with charge–charge interactions (*C*), hydrogen bonding (*D*), and hydrophobic interactions (*E*).

To dissect the molecular basis governing the ELKS/Rab6 interaction, we tried to solve the complex structure of ELKS1 and Rab6B by using crystallography. Using high-quality samples of ELKS1_RBD and Rab6B^Q72L^, we crystallized the ELKS1_RBD/Rab6B^Q72L^ complex and determined the complex structure at 2.04 Å resolution (Table S1). In the crystal, each asymmetric unit contains two ELKS1_RBD molecules and two Rab6B^Q72L^ molecules, which form two essentially identical complexes with a binding ratio of 1:1 between ELKS1_RBD and Rab6B^Q72L^ (Fig. S2*A*). In the complex structure (Fig. 2*A*), Rab6B^Q72L^ adopts the typical active GTPase conformation with a bound GTP molecule and ELKS1_RBD consists of two α-helices (αN and αC) to interact with the switch Ⅰ, switch Ⅱ, and interswitch regions of Rab6B^Q72L^. The two helices in ELKS1_RBD fold as a helical hairpin through interhelical hydrophobic interactions (Fig. S2*B*), which resembles the coiled-coil conformation and explains the monomer instead of dimer formation of this predicted coiled-coil region.

The binding of ELKS1_RBD to Rab6B^Q72L^ involves both polar and hydrophobic interactions. Specifically, four pairs of charged residues (E857_ELKS1_-R84_Rab6B_, E869_ELKS1_-K13_Rab6B_, D871_ELKS1_-R63_Rab6B_, and K905_ELKS1_-D49_Rab6B_) form salt bridges to stabilize the interaction between ELKS1 and Rab6B (Fig. 2*B*). The ITC-based analysis showed that the charge-reverse mutations, including D871R_ELKS1_, K905E_ELKS1_, D49R_Rab6B_, and R63E_Rab6B_, abolished the ELKS1_RBD/Rab6B^Q72L^ interaction (Fig. 2*C*). Among these charged residues, D871_ELKS1_ and K13_Rab6B_ are further involved in the hydrogen bond network connected by S868_ELKS1_ and Q65_Rab6B_ (Fig. 2*B*), which further strengthens the binding of ELKS1_RBD to Rab6B^Q72L^. Consistently, disrupting the hydrogen bond network by replacing either Q65 or S868 with an alanine dramatically decreases the binding affinity (Fig. 2*D*). In addition to the polar interactions, several hydrophobic residues in ELKS1_RBD, including L864, I867, T908, and M912, pack with the hydrophobic pocket formed by the switch II and interswitch regions of Rab6B (Fig. 2*B*). Also, A875, L876, and L879 in αN of ELKS1_RBD form a hydrophobic patch to accommodate L11 in Rab6B. Interfering with the hydrophobic interaction by mutating the hydrophobic residues to hydrophilic ones impairs the interaction between ELKS1_RBD and Rab6B^Q72L^ (Fig. 2*E*).

**Figure 3:**
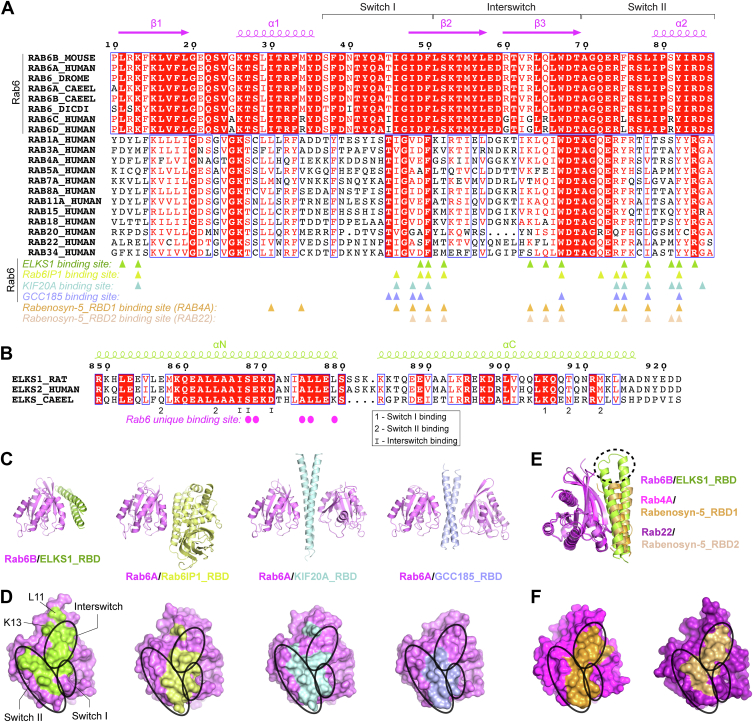
**Sequence and structural comparison of the Rab-binding modes of ELKS and other Rab effectors.***A*, amino acid sequence alignments of effector-binding regions in Rab proteins. The sequences of Rab6 proteins from mammals, *Drosophila*, *C. elegans*, and slime mold and those of other representative Rab family members from humans were aligned separately and then merged for comparison. Interface residues in Rabs that form contacts with their corresponding effectors were labeled with *triangles*. *B*, amino acid sequence alignment of the RBDs of ELKS proteins. Unique residues for Rab6 binding were indicated as *purple circles*. Residues involved in binding to the switch I, II, and interswitch regions of Rab6B are indicated. *C*, structural comparison of Rab6B/ELKS1_RBD to other Rab6/effector complexes, including the Rab6A/Rab6IP1 complex (PDB ID: 3CWZ), the Rab6A/KIF20A_RBD complex (5LEF), and the Rab6A/GCC185_RBD complex (3BBP). *D*, comparison of effector-binding surfaces on the Rab6 structures. *E*, superposition of the Rab6B/ELKS1_RBD structure with two Rab/Rabenosyn-5 structures (1Z0Q and 1Z0J). Compared to the two RBDs of Rabenosyn-5, ELKS1_RBD adopts a longer helical hairpin. The structural difference between them was highlighted by a *dashed ellipse*. *F*, comparison of Rabenosyn-5-binding surfaces on the Rab4A and Rab22 structures.

Notably, most of the interface residues in both ELKS1 and Rab6B are highly conserved in the ELKS and Rab6 family members (Fig. 3, *A* and *B*). However, the residue R63 in Rab6B is substituted with a glycine in Rab6C and Rab6D, which eliminates the R63-mediated salt bridging to ELKS1. Moreover, the residue G25 in Rab6B is substituted with alanine in Rab6C and Rab6D, leading to steric hindrance that interferes with GTP binding (Fig. S3) (27). Thus, the ELKS-binding mode described here is very likely to be shared by other Rab6 proteins, except for Rab6C and Rab6D.

### Structural comparison reveals the Rab6-binding specificity of ELKS1

Currently, the Rab6 structures in complex with some effectors, including Rab6IP1, GCC185, and KIF20A have been solved (7, 28, 29, 30). Despite the differences in the Rab6-binding regions of the three effectors (Fig. 3*C*), they cover a similar surface on Rab6, mainly formed by the switch Ⅰ and Ⅱ regions (Fig. 3, *A* and *D*). Compared to the other Rab6 effectors, ELKS1 occupies a larger surface on Rab6, encompassing not only the switch regions but also the interswitch region (Fig. 3, *A*, *C*, and *D*). Additionally, despite its coiled coil-like fold, ELKS1_RBD adopts a distinct binding orientation with Rab6, as compared to the coiled coils of KIF20A_RBD and GCC185_RBD (Fig. 3*C*). Nevertheless, ELKS1_RBD binds to Rab6 with an affinity in the μM range (Fig. 1*D*), similar to other Rab6 effectors (28, 30).

To understand the binding specificity of ELKS1_RBD for Rab6, we compared our structure with other Rab structures that have a bound effector with a similar helical hairpin conformation, including the Rab4A and Rab22 structures in complex with the two RBDs of Rabenosyn-5, respectively (Fig. 3*E*). Similar to ELKS1_RBD, the two RBDs of Rabenosyn-5 mainly bind to the switch II and interswitch regions of the Rab proteins (Fig. 3, *A* and *F*). However, compared to those in Rabenosyn-5, the helical hairpin of ELKS1_RBD extends further toward the N-termini of the Rab structures (Fig. 3*E*). The extended part of ELKS1_RBD interacts with a region outside the canonical effector binding site, specifically, the residues L11 and K13 in the β1-strand of Rab6B^Q72L^ (Fig. 2*B*), which are strictly conserved in Rab6 family members but not in other Rab members (Fig. 3*A*). Disrupting this interaction using the L11 E mutation in Rab6B^Q72L^ dramatically decreases the binding affinity of ELKS1_RBD (Fig. 2*E*). Thus, our structure explains the specific binding of ELKS proteins to Rab6 rather than other Rabs.

### ELKS1 forms protein condensates for accumulating Rab6 in cells

**Figure 4:**
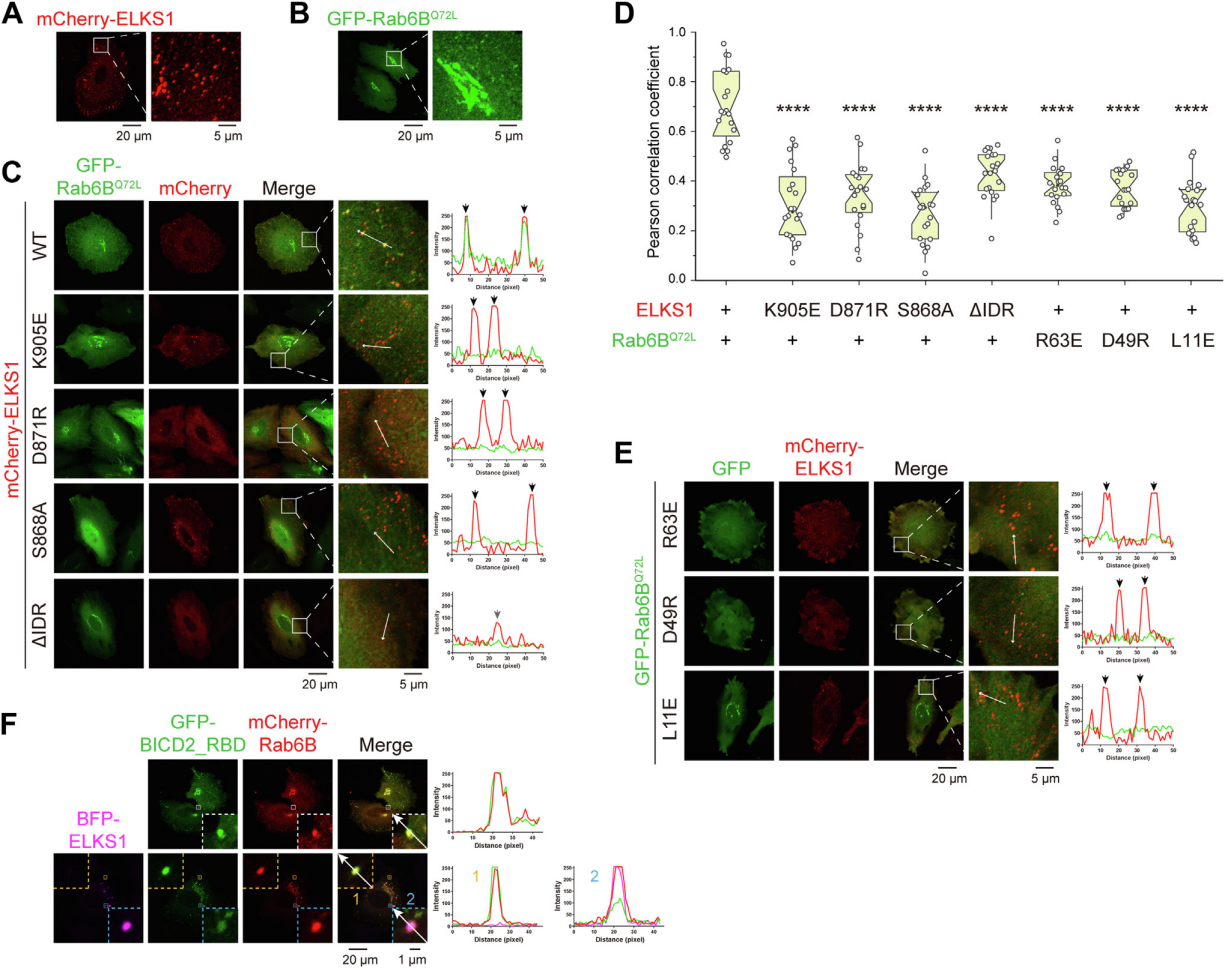
**ELKS1 accumulates Rab6B by forming cellular condensates.***A* and *B*, cell imaging of exogenous expressed mCherry-tagged ELKS1 (*A*) and GFP-tagged Rab6B^Q72L^ (*B*). The regions where ELKS1 or Rab6B can form puncta were selected as the ROIs, and the ROIs were *boxed* and enlarged. Scale bar was indicated at the *bottom*. *C*, cell imaging of exogenous co-expressed mCherry-tagged ELKS1 or its variants with GFP-tagged Rab6B^Q72L^. The regions where ELKS1 can form puncta were selected as the ROIs, and the ROIs were *boxed* and enlarged. Line analyses of fluorescent intensities were shown on the *right side*. Intensity peaks of ELKS puncta were indicated by *arrows*. *D*, Pearson correlation coefficients of GFP and mCherry signals were measured using the cell imaging data in (*C*) and (*E*). The whole cell fluorescence was used for analysis. Data were collected from 20 cells in each condition. Bars represent the means ± S.D. The unpaired Student’s *t* test analysis was used to define a statistically significant difference (∗∗∗∗*p* < 0.0001). *E*, cell imaging of exogenous co-expressed GFP-tagged Rab6B^Q72L^ or its variants with mCherry-tagged ELKS1. The regions where ELKS1 was able to form puncta were selected as the ROIs, and the ROIs were *boxed* and enlarged. Line analyses of fluorescent intensities were shown on the *right side*. Intensity peaks of ELKS punta were indicated by *arrows*. *F*, cell imaging analysis of the potential competition between ELKS1 and BICD2 for accumulating Rab6 by the co-expression of GFP-tagged BICD2_RBD and mCherry-tagged Rab6B^Q72L^ with or without BFP-tagged ELKS1. Two ROIs were boxed and enlarged in the condition with the expression of BFP-tagged ELKS1 to display different levels of BICD2_RBD in Rab6 enriched puncta formed with or without ELKS1. ROI, regions of interest.

ELKS1 forms many small puncta in cells (Fig. 4*A*) (31, 32) while Rab6 is mainly located in the Golgi apparatus and vesicles (Fig. 4*B*) (13, 15). However, overexpressing ELKS1 in the Rab6B^Q72L^-transfected HeLa cells drove the accumulation of Rab6B^Q72L^ in the ELKS1 puncta rather than the opposite (Fig. 4, *C* and *D*). To test whether the RBD in ELKS1 is essential for Rab6 recruitment, we mutated the Rab6-binding sites in ELKS1 according to our structural and biochemical findings. Although the ELKS1 mutants remained their punctate distribution in cells, they failed to accumulate Rab6B^Q72L^ in their puncta (Fig. 4, *C* and *D*). Likewise, mutations in Rab6B^Q72L^ that disrupt the ELKS1 binding diminished the accumulation of Rab6B^Q72L^ in the ELKS1 puncta (Fig. 4, *D* and *E*), indicating that ELKS1 can recruit and accumulate Rab6 in an RBD-dependent manner. The D49R and R63E mutants of Rab6B^Q72L^ were observed to accumulate modestly in the Golgi apparatus (Fig. 4*E*), likely due to the involvement of D49 and R63 in Rab6’s binding to Golgi-related effectors, such as Rab6IP1 and GCC185 (28, 29). Interestingly, although the L11E mutant of Rab6B^Q72L^ failed to be recruited to the ELKS1 puncta, it still retained its localization to the Golgi (Fig. 4, *D* and *E*). The above observations confirm our structural finding that although the ELKS-binding surface on Rab6 largely overlaps with other effector-binding surfaces, ELKS proteins possess a unique mode of binding to Rab6 (Fig. 3*D*). Notably, as L11E did not affect the Golgi localization of Rab6B^Q72L^, this mutation may be used to study Rab6 functions specifically related to ELKS in the future.

Bicaudal D1/D2 (BICD1/2) proteins are Rab6 effectors that link Rab6-bound membranes with cytoskeletal motors, including dynein and kinesins (33, 34, 35). Consistently, we observed robust colocalization of Rab6B^Q72L^ and BICD2_RBD when the two proteins were co-expressed (Fig. 4*F*). However, co-transfecting ELKS1, BICD2_RBD, and Rab6B^Q72L^ into HeLa cells, we found that BICD2_RBD and ELKS1 mutually limited each other from entering their puncta, despite their puncta both accumulating Rab6B^Q72L^ (Fig. 4*F*). As BICD proteins have a sub-μM binding affinity to Rab6 that is much stronger than ELKS1’s binding to Rab6 (Fig. 1*D*) (36), it raises a question about how ELKS1 can effectively compete with BICDs for binding to Rab6B^Q72L^.

Considering ELKS proteins have the propensity to undergo liquid-liquid phase separation (LLPS) (31, 32, 37), we speculated that ELKS1 forms protein-rich condensate in cells *via* LLPS, which increases the local concentration of ELKS1 and thereby facilitates the acquisition of Rab6. To test this hypothesis, we designed a ELKS1 mutant that lacks the N-terminal IDR to disrupt the LLPS capability of ELKS1, as this region has been shown to be essential for LLPS in ELKS proteins (32). Indeed, rather than forming puncta, ELKS1^ΔIDR^ showed a predominantly diffused distribution in the transfected cells (Fig. S4). A small number of puncta weakly enriched with ELKS1^ΔIDR^ (Fig. S4), likely due to its capability to associate with endogenous ELKS1 protein *via* the coiled coil-mediated dimer formation (32). Nevertheless, these ELKS1^ΔIDR^-enriched puncta failed to accumulate Rab6B^Q72L^ (Fig. 4, *C* and *D*), although ELKS1^ΔIDR^ contains the intact RBD, suggesting that the condensed ELKS1 enhances the acquisition of Rab6 from other Rab6 effectors.

### The ELKS1 condensate recruits Rab6B-coated liposomes

**Figure 5:**
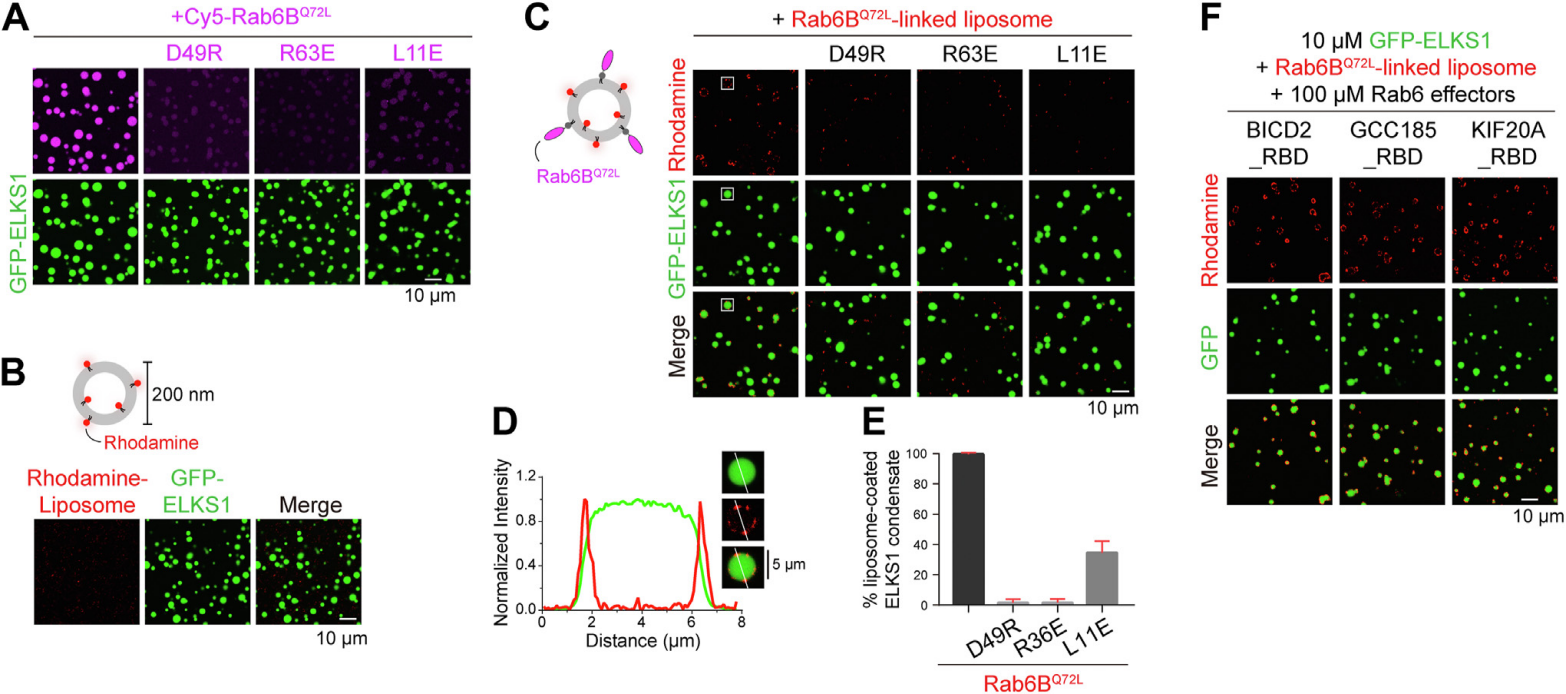
**Condensed ELKS1 recruits Rab6B-coated liposomes.***A*, fluorescence imaging of phase-separated GFP-ELKS1 in accumulating Rab6B^Q72L^. Rab6B^Q72L^ was highly enriched in the droplets formed by ELKS1 but not its Rab6-binding deficient mutations (D49R, R63E, and L11E). Rab6B^Q72L^ and ELKS1 were mixed at a 1:1 ratio at 10 μM concentrations. Rab6B was labeled with Cy5 at 5% level. *B*, fluorescence imaging of phase-separated GFP-ELKS1 in mixing with Rhodamine-labeled liposomes. *C*, fluorescence imaging of phase-separated GFP-ELKS1 in mixing with Rab6B-linked liposomes. Rab6B^Q72L^ and its ELKS-binding deficient mutants were chemically linked to maleimide lipid by C-terminal cysteine and quenched by DTT. Rab6B^Q72L^-linked liposomes accumulated on the surface of ELKS1 droplets, which was disrupted by the ELKS-binding deficient mutations. A region of interest was *boxed* and enlarged as shown in (*D*). *D*, the zoom-in view of the boxed region in (*C*) shows that the Rab6B^Q72L^-liposome accumulates on the ELKS1 droplet surface. Line analysis of fluorescent intensities was shown below. *E*, quantification of the percentage of ELKS1 condensates coated with Rab6B^Q72L^-liposome. Eight views were selected for each condition and the number of liposome-coated droplets over the number of total droplets was calculated for each view. *F*, fluorescence imaging of the mixture of phase-separated GFP-ELKS1 and Rab6B-linked liposomes in the presence of Trx-tagged proteins of other Rab6 effectors, including BICD2_RBD, GCC185_RBD, and KIF20A_RBD.

Since ELKS1 was reported to capture Rab6-coated vesicles in neurons and nonneuronal cells (14, 15, 38), we next asked whether the ELKS1 condensate can recruit Rab6-coated vesicles. By using purified full-length ELKS1 protein, we were able to generate ELKS1 condensates (Fig. S5, *A* and *B*) similar to those previously reported using the N-terminal fragment (32). By the addition of Rab6B^Q72L^ to the phase-separated ELKS1, we found that Rab6B^Q72L^ was highly accumulated in the ELKS1 condensate (Fig. 5*A*). However, the mutations in Rab6B^Q72L^ that disrupt its binding to ELKS1 led to a depletion of Rab6B^Q72L^ accumulation (Fig. 5*A*), indicating the crucial role of the RBD-dependent interaction in recruiting Rab6 to the ELKS1 condensate.

As ELKS proteins play a critical role in the targeting of secretory vesicles to specific areas of the plasma membrane and melanosomes (15, 39), we sought to investigate whether the membraneless condensed phase of ELKS1 could enrich membranous Rab6-coated vesicles. To study the interaction between the Rab6-coated vesicles and ELKS condensates, we first prepared rhodamine-labeled liposomes with a diameter of approximately 200 nm to mimic transported vesicles. The rhodamine-labeled liposomes did not associate nonspecifically with the ELKS1 condensate, as indicated by no rhodamine-signal in phase-separated ELKS1 droplets in the ELKS1/liposome mixture (Fig. 5*B*). Next, we chemically linked Rab6B^Q72L^ to rhodamine-liposomes and mixed them with ELKS1 droplets (Fig. 5*C*). Interestingly, the Rab6B^Q72L^-linked liposomes did not enter the ELKS1 condensate but instead accumulated on the surface of ELKS1 droplets (Fig. 5, *C* and *D*). This observation indicates that the condensed ELKS1 phase can indeed capture vesicles, but the ELKS1 condensate is not immersible for membranous vesicles. In contrast, the liposome accumulation on the ELKS1 condensate was blocked by the D49R, R63E, and L11E mutations in Rab6B^Q72L^ (Fig. 5, *C* and *E*), confirming that the liposome recruitment by the ELKS1 condensate is dependent on Rab6 binding. Moreover, the inactive mutant Rab6B^T27N^-coated liposomes showed no accumulation on the droplet surface (Fig. S5*C*), further highlighting the importance of the specific binding of ELKS1 to the active form of Rab6 in vesicle recruitment.

To further validate the competition between the condensed ELKS1 and other Rab6 effectors in the dilute phase for recruiting Rab6-coated vesicles, we added the purified RBDs of BICD2, GCC185, and KIF20A into the mixture of the Rab6B^Q72L^-coated liposome and phase-separated ELKS1. Although having a 10-fold higher protein concentration in solution, these RBDs showed little effect on the accumulation of Rab6B^Q72L^-coated liposomes on the ELKS1 droplets (Fig. 5*F*), confirming the capability of the condensed ELSK1 in acquiring Rab6-coated vesicles from other Rab6 effectors.

### ELKS1-mediated accumulation of Rab6 promotes the NPY secretion

**Figure 6:**
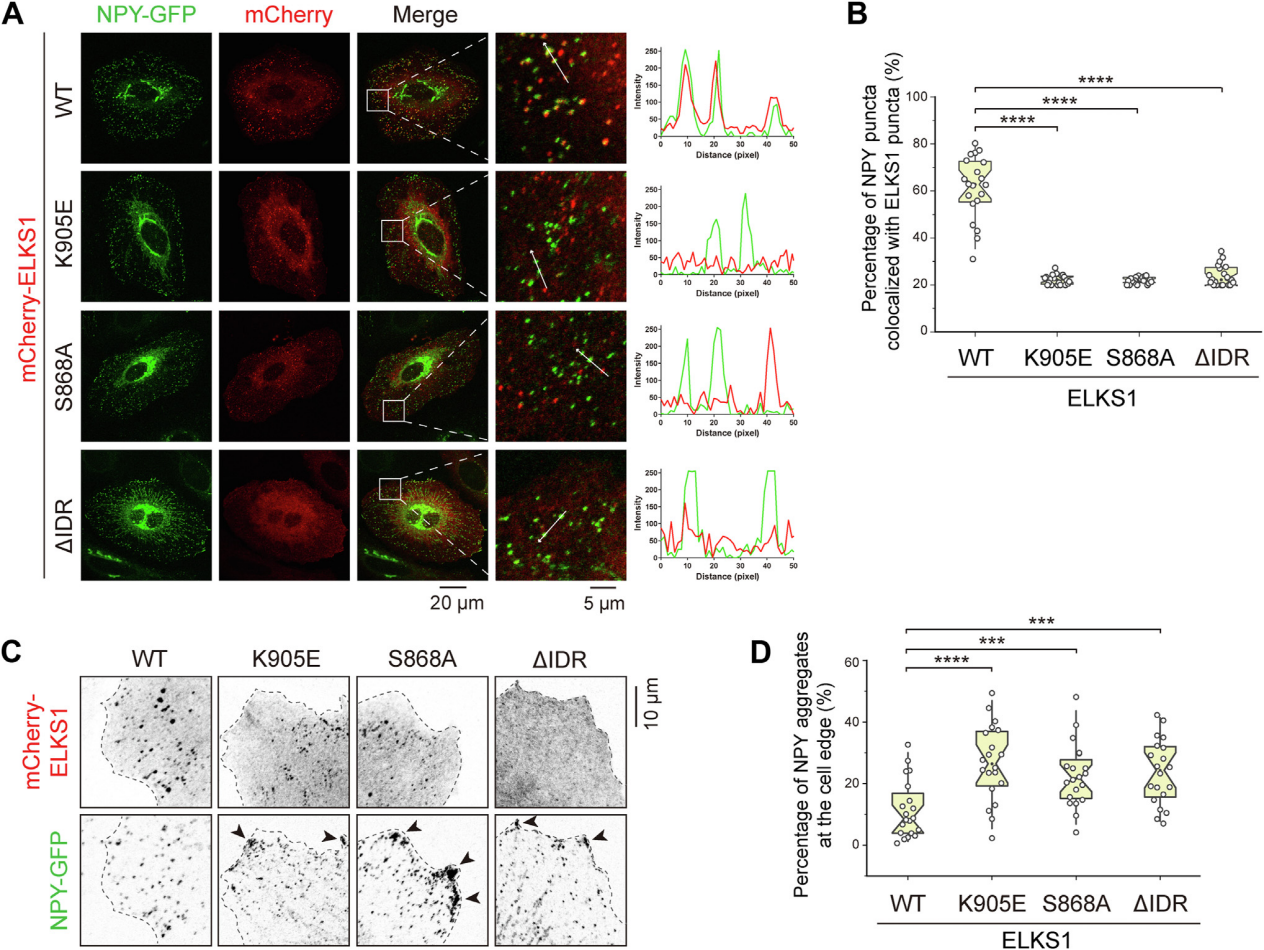
**ELKS1 promotes NPY secretion by recruiting Rab6.***A*, cell imaging of GFP-tagged NPY co-expressed with mCherry-tagged ELKS1 or its variants. The regions where the NPY-vesicles were relatively clear as the ROIs, and the ROIs were *boxed*, enlarged, and aligned at the *right* of each merged image. Line analysis of fluorescent intensities is shown on the *right panel*. *B*, quantifications of colocalization of the NPY and ELKS1 puncta in (*A*). Data were collected from 20 cells in each condition. Bars represent the means ± S.D. The unpaired Student’s *t* test analysis was used to define a statistically significant difference (∗∗∗∗*p* < 0.0001). *C*, representative cell images of peripherical NPY vesicles in the different conditions with the overexpressed mCherry-tagged ELKS or its variants. *D*, quantifications of peripherical NPY aggregates in (*C*). Data were collected from 20 cells in each condition. Bars represent the means ± SD. The unpaired Student’s *t* test analysis was used to define a statistically significant difference (∗∗∗*p* < 0.001; ∗∗∗∗*p* < 0.0001). ROI, regions of interest.

Since ELKS1 may surpass other Rab6 effectors for binding to Rab6 by forming condensates, we speculated that ELKS1 unloads Rab6-coated vesicles to the releasing site by competing with Rab6 effectors in the transport machine. As ELKS proteins and Rab6 are known to be involved in the secretion of neuropeptide Y (NPY) (15), we decided to examine the impact of ELKS1-mediated accumulation of Rab6 on NPY secretion. To assess this, we overexpressed GFP-tagged NPY as an indicator to monitor NPY exocytosis in HeLa cells. Our results showed that most NPY vesicles colocalized with ELKS1 but not its Rab6-binding deficient mutants (K905E and S868A) (Fig. 6, *A* and *B*), indicating that the RBD-dependent binding of ELKS1 to Rab6 is required for the recruitment of NPY vesicles to the secretion site enriched with ELKS1. Importantly, NPY vesicles accumulated at the cell edge when the K905E or S868A mutant of ELKS1 was overexpressed, which was not observed in the cells overexpressing the wild-type ELKS1 (Fig. 6, *C* and *D*). These results suggest that the accumulation of Rab6 in the ELKS puncta is critical for the efficient secretion of NPY vesicles.

Next, we asked whether the LLPS of ELKS1 contributes to the NPY secretion. To address this question, we used the ELKS1^ΔIDR^ mutant in the NPY secretion assay. As shown in Figure 6, *A* and *B*, ELKS1^ΔIDR^ was unable to colocalize with NPY vesicles, despite retaining its RBD for Rab6 binding, likely because ELKS1^ΔIDR^ cannot effectively compete with other Rab6 effectors in NPY vesicles for Rab6 binding. Consequently, ELKS1^ΔIDR^ failed to promote the NPY secretion and led to the NPY vesicle aggregation at the cell edge (Fig. 6, *C* and *D*), confirming the critical role of IDR-dependent LLPS in boosting Rab6 binding for ELKS1-mediated vesicle capturing and exocytosis.

## Discussion

Although intracellular vesicle trafficking has been studied for decades, the molecular mechanism underlying the unloading of transporting vesicles from the transport machine upon arrival at their destination remains poorly understood. As master organizers in vesicle trafficking, Rab GTPases, particularly Rab6, play a central role in regulating vesicle mobility, including transport, tethering, docking, and fusion (15, 38). In this study, we elucidated the structural basis of the unique Rab6-binding mode of ELKS proteins. The high-resolution structure of the ELKS1_RBD/Rab6B complex allows us to design specific mutations to disrupt the interaction between ELKS and Rab6. Using these mutations, we confirmed the ELKS1/Rab6 interaction is critical for the secretion of NPY transported by Rab6-marked vesicles. Additionally, our findings suggest that ELKS1 enhances its capability to capture Rab6-coated vesicles by forming condensates.

**Figure 7:**
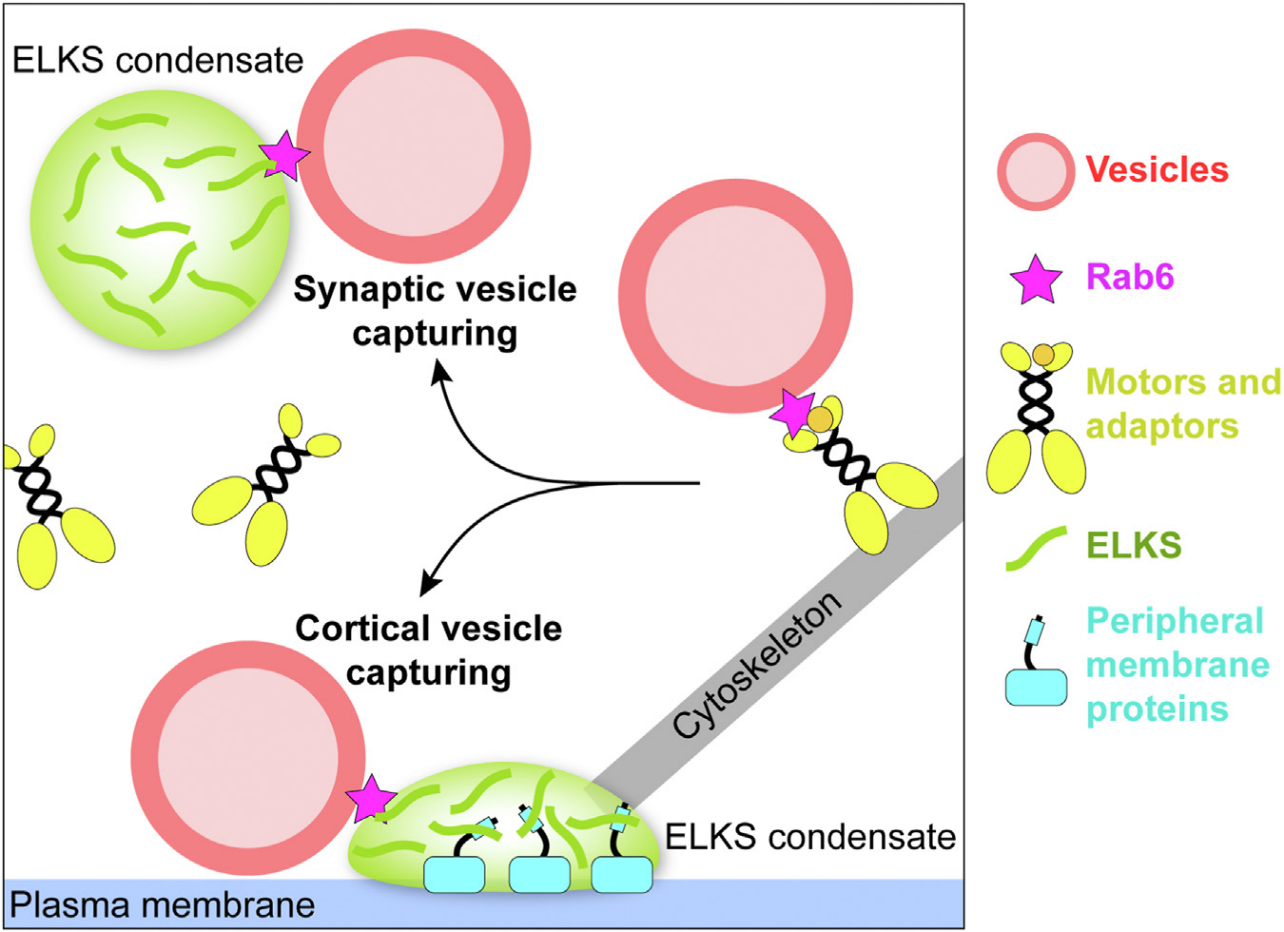
**A proposed model of the phase-separated ELKS in capturing and unloading Rab6-coated vesicles from vesicle-transporting motors in the presynapse for vesicle deposition and in the cell cortex for vesicle secretion.** The ELKS condensates can be attached to the plasma membrane aided by peripheral membrane proteins, like LL5β. After unloading cargos, motors may be recycled for the next run of transport.

The question of how ELKS proteins capture and retain Rab6 vesicles specifically in the crowded environment of the vesicle targeting sites, such as the presynaptic region that fills with synaptic vesicles, endosomes, and other lipid-wrapped organelles (22, 40, 41, 42, 43), has remained unsolved given that Rab6 binds to ELKS with a relatively low binding affinity (Fig. 1, *D* and *E*) and Rab proteins attached to secretory vesicles have small protein copy numbers (44). Based on our findings, we proposed a model for ELKS1-mediated vesicle capturing (Fig. 7). In this model, Rab6-positive vesicular cargos are transported by cytoskeletal motors, with Rab6 acting as the molecular linkage between the vesicles and the motor/adaptor complex. As the transported vesicles approach their destination site (*e.g.*, synaptic vesicles for deposition in the presynapse (3, 45) and insulin vesicles for releasing in the cell cortex (25)), ELKS proteins trap the vesicles near the site by competing with adaptors such as BICD1/2 for binding to Rab6 and thereby unload the vesicle from the transport machine. A plausible mechanism to accomplish this process is by forming ELKS condensates *via* LLPS, which may locally concentrate ELKS to ∼1 mM (32) for the efficient recruitment of Rab6 vesicles. Certain membrane-anchored proteins, such as LL5β (24), may aid in localizing ELKS to the plasma membrane, thereby enabling the vesicles attached to the ELKS condensate to approach the membrane for docking and fusion.

The ELKS-mediated vesicle capturing in neurons may involve some other ELKS binding partners in addition to Rab6, such as liprin-α and RIM (Fig. 1*A*) (20, 46), which are also key scaffold proteins in presynapse (6). Liprin-α was reported to regulate kinesin motors for synaptic vesicle trafficking (47), while RIM proteins were found to promote the synaptic vesicle tethering to the presynaptic terminal (48). Thus, the binding of liprin-α and RIM to ELKS may work together with the ELKS/Rab6 interaction to synergistically enhance the ELKS-mediated capture and release of synaptic vesicles.

Emerging evidence has shown that membraneless condensates can interact with membrane-bound organelles to modulate their organization or facilitate the trafficking and storage of organelles (49, 50). In the synapse, protein condensates have been discovered to cluster synaptic vesicles, confining their spatially, and to tether them to presynaptic membrane for quick fusion in response to stimuli (51, 52, 53). Protein condensates have also been found to facilitate the formation of diverse types of vesicles and organelles from the endoplasmic reticulum (ER) tubules (54, 55, 56). Our study here adds a new scenario to the usage of membraneless condensates in vesicle trafficking, by demonstrating the role of ELKS-mediated condensates in the specific capturing of Rab6-positive vesicles at the cell cortex. Recently, GCC185 and several other golgin family proteins were reported to form condensates (57). Considering the interactions between golgins and Rabs, the golgin-formed condensates are likely to play a role in the golgin-mediated membrane capturing and tethering.

## Experimental procedures

### Expression constructs and site-directed mutagenesis

DNA encoding sequences of mouse Rab6B (GenBank: NM_173781.4) were amplified from a mouse brain cDNA library. The full-length (1–208) or GTPase domain (8–176) of Rab6B was cloned into the modified pET-32a vector with an N-terminal Trx-His_6_ tag and an HRV-3C protease cutting site. Plasmids containing rat ELKS1 (GenBank: AY174115.1) were kind gifts from Prof. Mingjie Zhang. The full-length ELKS1 was first cloned into a modified pETL7 vector with an N-terminal His_6_-MBP-GFP tag followed by a TEV-protease cutting site that is kindly provided by Prof. Pilong Li and then His_6_-MBP-GFP-ELKS1 was subcloned into the pCAG vector with an N-terminal FLAG tag. The ELKS1 fragments used for biochemical and structural characterizations were subcloned into the modified pET-32a vector. The Rab6 binding domains (RBDs) of human BICD2 (aa 662–804), KIF20A (aa 603–665), and GCC185 (aa 1547–1612) were amplified from the cDNA library and cloned into the modified pET-32a vector. DNA encoding sequences of human NPY (GenBank: NM_000905.3) were commercially synthesized by Tsingke Biotech and cloned into the pEGFP-N1 vector. All point mutations were created using a site-directed mutagenesis kit and confirmed by DNA sequencing.

### Protein expression and purification

For LLPS assay, the full-length ELKS1 was expressed in HEK 293F suspension cells (Thermo Fisher Scientific), cultured in Freestyle 293 medium (OPM-293 CD05 Medium) at 37 °C supplied with 5% CO_2_ and 80% humidity. When the HEK 293F cell density reached 2.0 × 10^6^ cells per ml, the cells were transiently transfected with the expression plasmids and polyethylenimines (PEIs) (Polysciences). For transfection, approximately 0.5 mg ELKS1 plasmids were pre-mixed with 1 mg PEIs in 50 ml fresh medium for 15 min and then 50 ml mixture was added to 500 ml cell culture. After 72 h culture, cells were collected at 4 °C and lysed with 50 mM Tris pH 7.5, 500 mM NaCl, 1 mM EDTA, 0.5% Triton X-100, and 1 × protease inhibitor cocktail. The ELKS1 protein was purified by anti-FLAG affinity chromatography and competitively eluted with buffer containing 100 to 500 μg FLAG peptide (DYKDDDDK) and followed by size-exclusion chromatography (Superdex 6 Increase, GE healthcare) with a buffer containing 50 mM Tris pH 7.5, 100 mM NaCl, and 2 mM DTT.

Trx-His_6_ fusion proteins were expressed in BL21(DE3) *E. coli* cells in LB medium after induction with 500 μM isopropyl-β-d-thiogalactopyranoside at 16 °C overnight. Cells were collected at 4 °C and disrupted with high pressure in 50 mM Tris pH 8, 500 mM NaCl, 10 mM Imidazole, 1 × protease inhibitor cocktail, and PMSF. Fusion proteins were purified by Ni^2+^-NTA affinity chromatography and followed by size-exclusion chromatography (Superdex-200, GE healthcare) in 50 mM Tris pH 7.5, 100 mM NaCl, 2 mM MgCl_2_, and 1 mM DTT. To prepare the Rab6B^Q72L^ and ELKS_RBD fragments for crystallization, the N-terminal His_6_-Trx tags were cleaved by HRV 3C protease and removed by size-exclusion chromatography (Superdex-75, GE healthcare).

### Crystallography

Rab6B^Q72L^ and ELKS1_RBD were mixed with a 1:1 ratio and incubated overnight at 4 °C in 50 mM Tris pH 7.5, 100 mM NaCl, 2 mM MgCl_2_, 1 mM DTT, and 1 mM GTP. For crystallization, the complex was purified on Superdex 75 16/60 and concentrated at 15 mg/ml. Crystals were obtained by the sitting drop vapor diffusion method at 16 °C. To set up a sitting drop, 1 μl of concentrated protein solution was mixed with 1 μl of crystallization solution. The crystals of Rab6B^Q72L^ and ELKS1_RBD complex were grown in the conditions containing 0.1 M HEPES pH 7.5 and 42 % v/v Polyethylene glycol 200. Before X-ray diffraction experiments, crystals were soaked in the crystallization solutions containing additional 30% v/v glycerol for cryoprotection. Diffraction data were collected at the Shanghai Synchrotron Radiation Facility. Data were processed and scaled using HKL2000 software (58).

### Structure determination and analysis

The crystal structures of Rab6B^Q72L^ and ELKS1_RBD complex were determined by molecular replacement in PHASER (59) using the Rab6B^Q72L^ apo structure (PDB ID: 2FFQ) as the search model. The structural models were refined in PHENIX (60) and adjusted in COOT (61). The model quality was checked by MolProbity (62). The final refinement statistics are listed in Table S1. All structure figures were prepared by using PyMOL (https://www.pymol.org).

### Analytical gel filtration chromatography

Analytical gel filtration chromatography was carried out on an ÄKTA pure system (GE Healthcare). Protein samples were loaded with 100 μl sample loop into a Superdex 200 Increase 10/300 Gl column (GE Healthcare) and equilibrated with a buffer containing 50 mM Tris pH 7.5, 100 mM NaCl, 2 mM MgCl_2_, and 1 mM DTT.

### Multi-angle light scattering analysis

A miniDAWN TREOS (Wyatt Technology Corporation) coupled with an ÄKTA pure system (GE Healthcare) was used for molar mass measurement. The procedure follows the protocol used for analytical gel filtration analysis.

### Isothermal titration calorimetry analysis

Isothermal titration calorimetry (ITC) experiments were carried out on MicroCal PEAQ-ITC (Malvern Panalytical) at 25 °C. All proteins were dissolved in a buffer containing 50 mM Tris pH 7.5, 100 mM NaCl, and 2 mM MgCl_2_. Each titration point typically is consisted of injecting 3 μl aliquots of one protein (400 μM) in the syringe into its binding protein (40 μM) in the cell. A time interval of 150 s between two titration points was used to ensure the complete equilibrium of each titration reaction.

### Protein fluorescence labeling

Cy5 NHS ester (AAT Bioquest) was dissolved by DMSO at a stock concentration of 5 mM. Purified proteins were exchanged to a buffer containing 100 mM NaHCO_3_ pH 8.3, 100 mM NaCl, and 2 mM DTT. Fluorophores were mixed with the corresponding proteins in a 1:1 ratio and incubated at room temperature for 1 h. The reaction was quenched by adding 200 mM Tris pH 8.2. To remove the unlabeled fluorophores, the labeled proteins were exchanged into a buffer containing 50 mM Tris pH 8.2, 100 mM NaCl, and 2 mM DTT using a HiTrap desalting column. Fluorescence labeling efficiency was measured by Nanodrop 2000 (ThermoFisher). For imaging assays, fluorescently labeled proteins were added to the corresponding unlabeled proteins in the same buffer with the final molar ratio of 1:20.

### Liposome preparation

Liposomes were prepared with commercially available lipid components from Avanti Polar Lipids. A mixture of lipids was dissolved in chloroform at (82.4% POPC, 15% POPS, 2.5% 18:1 PE MCC, and 0.1% rhodamine PE; Avanti). The lipids were then dried under a constant stream of nitrogen and desiccated in a vacuum overnight. The lipid film was resuspended with a buffer containing 30 mM Hepes, pH 7.4, 50 mM KCl, 2 mM MgCl_2_, and 1 mM EGTA and passed 25 times through an extruder containing a 200-nm pore-size polycarbonate filter (Avanti). A 3:1 ratio of Rab6B^Q72L^ to maleimide lipids was then mixed and incubated overnight. Next, DTT was added to a final concentration of 10 mM to quench the reaction, and the liposomes were pelleted at 160,000*g* for 20 min to remove unlabeled protein. The pellet was finally washed and resuspended with buffer.

### LLPS assays

Stock proteins were centrifuged at ∼20,000*g* for 10 min at 4 °C to remove any precipitations and diluted to designed concentrations with a dilution buffer containing 50 mM Tris pH 7.5, 100 mM NaCl, and 2 mM DTT. To induce LLPS, MBP-GFP tagged ELKS1 were treated with TEV protease for ∼20 to 30 min and then dripped onto the 384-well glass bottom plates (P384-1.5H-N, Cellvis). The confocal images were taken by A1R (Nikon) with 100× oil objective lens. To monitor proteins in different samples under light microscopes, the unlabeled corresponding proteins were premixed with the fluorophore-labeled proteins. The volume of phase separated protein solution adding to the well was ∼5 μl. For the treatment with liposomes, each protein with an assigned concentration and liposomes were mixed in tubes for 3 min and then dropped onto the 384-well plates for imaging. During imaging, the 384-well plates were sealed by plastic stickers to reduce solution evaporation. Image fluorescence intensities were analyzed by the ImageJ/Fiji software.

### Cell culture, transfection, and fluorescence imaging

HeLa cells were maintained in Minimum Essential Medium Eagle supplemented with 10% fetal bovine serum, 1% penicillin-streptomycin solution, and 1% non-essential amino acids. The cells were cultured at 37 °C in an incubator with 5% CO_2_. Transfections of indicated plasmids were performed with Lipofectamine 3000 Transfection Reagent (ThermoFisher) according to the manufacturer’s instructions, and the cells were transferred to fibronectin (12.5 μg/ml)-coated coverslips at 24 h after transfection. After 12 h, the cells were fixed with 4% paraformaldehyde for 15 min at 37 °C. After washing with the PBS buffer for three times, the cells were treated with 0.1% Triton X-100 for 10 min at room temperature and blocked in 2% bovine serum albumin. The cells were visualized with 100× objective using a Nikon A1R Confocal Microscope. Image pro plus software was used for line analysis and NIS-Elements AR Analysis software was used for co-localization analysis.

### Quantification and statistical analysis

Statistical parameters including the definitions and exact values of n (*e.g.*, number of experiment replications, number of cells, number of droplets, etc.) are reported in the figures or corresponding legends. All data were expressed as mean ± SD; ns, not significant; ∗*p* < 0.05, ∗∗*p* < 0.01, ∗∗∗*p* < 0.001, and ∗∗∗∗*p* < 0.0001 using unpaired Student’s *t* test. ITC titration data were analyzed using the program MicroCal PEAQ-ITC Analysis. MALS data were analyzed in program ASTRA 6.1.2 (Wyatt Technology Corporation). Statistical data were conducted in GraphPad Prism 6 software.

## Data availability

Atomic coordinates and structure factor amplitudes for the complex structure of ELKS1_RBD/Rab6B^Q72L^ have been deposited in the Protein Data Bank (https://www.wwpdb.org/) under the accession code 8IJ9. All other relevant data are available from the authors.

## Supporting information

This article contains supporting information.

## Conflict of interest

The authors declare no competing financial interests.
