# Supplementary material for: Structural basis of ELKS/Rab6B interaction and its role in vesicle capturing enhanced by liquid-liquid phase separation
Source: J Biol Chem. 2023 May 11;299(6):104808. doi: 10.1016/j.jbc.2023.104808 (PMC10267580; doi:10.1016/j.jbc.2023.104808)
Supplement: Supporting Figures S1–S5 and Table S1 [file mmc1.pdf]

Structural basis of ELKS/Rab6B interaction and its role in vesicle capturing enhanced by liquid-liquid phase separation

Gaowei Jin[1,2,4], Leishu Lin[1,2,4], Kaiyue Li[1,2], Jiashan Li[1,2], Cong Yu[2,3*], Zhiyi Wei[1,2*]

[1]Brain Research Center, Southern University of Science and Technology, Shenzhen, Guangdong 518055, China.

[2]School of Life Sciences, Southern University of Science and Technology, Shenzhen, Guangdong 518055, China.

[3]Guangdong Provincial Key Laboratory of Cell Microenvironment and Disease Research, and Shenzhen Key Laboratory of Cell Microenvironment, Shenzhen, Guangdong, China 518055.

[4]These authors contributed equally to this work.

Running title: ELKS captures Rab6 vesicles aided by phase separation

*Correspondences: (weizy@sustech.edu.cn) (Z.W.) and (yuc@sustech.edu.cn) (C.Y.)

Supporting information

Table S1. Statistics of data collection and structure refinement.

FIGURES

Figure S1. Analytical gel filtration analysis of the interaction between ELKS2 and Rab6B.
Figure S2. Structure analysis of the ELKS1_RBD/Rab6B^{Q72L} complex.
Figure S3. Structural analysis of the GTP-binding pocket in Rab6 proteins.
Figure S4. Imaging analysis of cells transfected with mCherry-ELKS1$^{\Delta IDR}$ showing its predominantly diffused distribution.
Figure S5. Liquid-liquid phase separation analysis of ELKS1.

Table S1. Statistics of data collection and structure refinement.

**Data collection**	
Space group	$P\ 2_1$
Cell dimensions	
a, b, c (Å)	49.193, 93.227, 53.077
α, β, γ (°)	90, 103.74, 90
Resolution (Å)	50–2.04 (2.15–2.04)
R_{merge}[a]	0.085 (0.327)
$I/\sigma I$	7.2 (2.4)
$CC_{1/2}$[b]	0.989 (0.800)
Completeness (%)	90.2 (61.6)
Redundancy	2.2 (1.8)
**Refinement**	
Resolution (Å)	50–2.04 (2.12–2.04)
No. reflections	26635 (1676)
R_{work} / R_{free}[c]	0.172 (0.213) / 0.225 (0.324)
No. atoms	
Protein	3822
Ligand/ion	76
Water	226
Mean B (Å)	
Protein	32.7
Ligand/ion	23.4
Water	31.3
r.m.s. deviations	
Bond lengths (Å)	0.010
Bond angles (°)	1.13
All-atom clashscore	1.66
Ramachandran analysis	
Favored region (%)	97.85
Allowed region (%)	2.15
Outliers (%)	0

The numbers in parentheses represent values for the highest resolution shell.

[a]$R_{merge} = \sum|I_i - I_m|/\sum I_i$, where Ii is the intensity of the measured reflection and I_m is the mean intensity of all symmetry related reflections.

[b]$CC_{1/2}$ is the correlation coefficient of the half datasets.

[c]$R_{work} = \Sigma||F_{obs}| - |F_{calc}||/\Sigma|F_{obs}|$, where F_{obs} and F_{calc} are observed and calculated structure factors. $R_{free} = \Sigma_T||F_{obs}| - |F_{calc}||/\Sigma_T|F_{obs}|$, where T is a test data set of about 4-5 % of the total reflections randomly chosen and set aside prior to refinement.

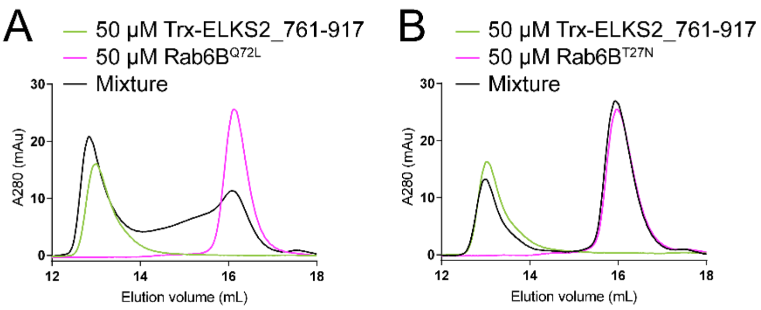

Figure S1. Analytical gel filtration analysis of the interaction between ELKS2 and Rab6B.

(A) Analytical gel filtration chromatography showing the interaction between ELKS2 and the active form of Rab6B.

(B)Analytical gel filtration chromatography showing no detectable binding between ELKS2 and the inactive form of Rab6B.

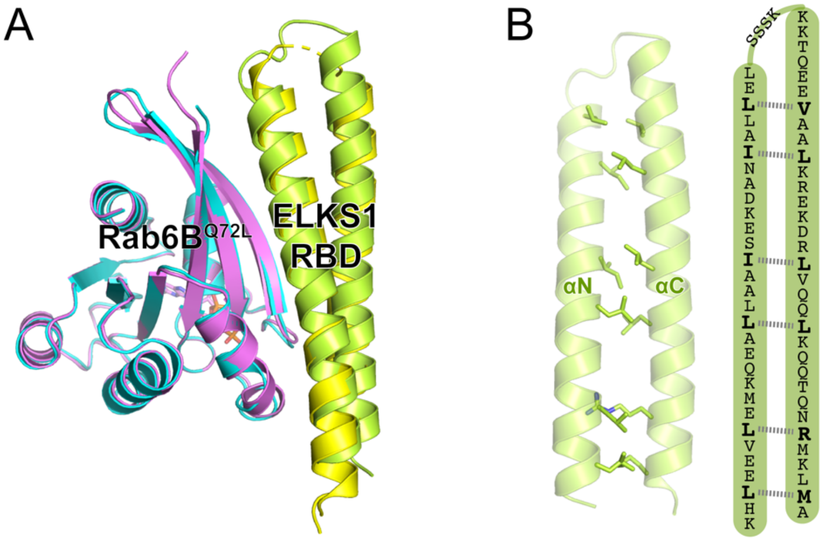

Figure S2. Structure analysis of the ELKS1_RBD/Rab6B^{Q72L} complex.

(A) Structural overlapping of the two Rab6B/ELKS1_RBD complexes in one asymmetric unit.

(B) The interhelical hydrophobic interactions in ELKS1_RBD. The residues that are involved the hydrophobic interactions were showed as sticks in the left panel and highlighted in the right panel. The close contacts between the residues were indicated as dashed lines in the right panel.

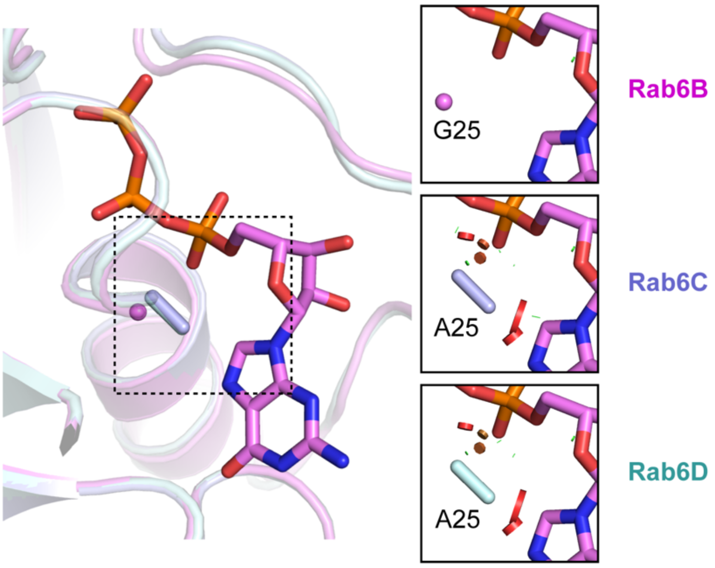

Figure S3. Structural analysis of the GTP-binding pocket in Rab6 proteins.

The AlphaFold2-predicted structures of Rab6C and Rab6D (https://alphafold.ebi.ac.uk) were aligned to our Rab6B structure. The atomic clashes between the marked residues and the bound GTP molecule were indicated by cylinders in the right panels.

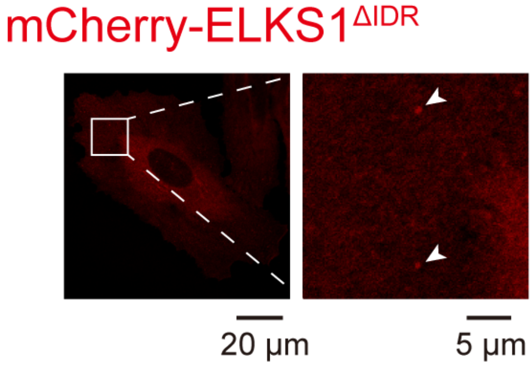

Figure S4. Imaging analysis of cells transfected with mCherry-ELKS1$^{\Delta IDR}$ showing its predominantly diffused distribution.

A small number of ELKS1$^{\Delta IDR}$ puncta with low fluorescent intensity can be also observed.

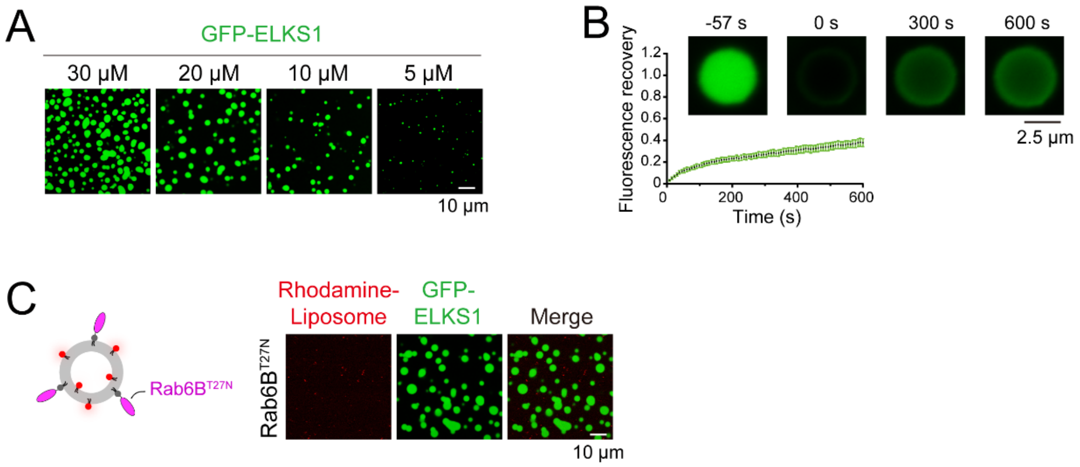

Figure S5. Liquid-liquid phase separation analysis of ELKS1.

(A) Fluorescence images showing that the full-length protein of ELKS1 undergoes LLPS in a concentration-dependent manner.

(B) The recovery curve of the GFP fluorescence signals in the condensed phase of ELKS1 after photobleaching. The concentration of protein in this assay was 10 μM. The curve represents the averaged signals from 3 droplets with similar size. Data are presented as mean ± SD.

(C) Fluorescence imaging of phase separated GFP-ELKS1 in mixing with Rab6B^{T27N}-linked liposomes. Rab6B^{T27N} was chemically linked to maleimide lipid by C-terminal cysteine and quenched by DTT. Rab6B^{T27N}-linked liposomes labeled with rhodamine dye were randomly distributed outside ELKS1 droplets.
